# Supplementary figures and images for: Conserved antigen structures and antibody-driven variations on foot-and-mouth disease virus serotype A revealed by bovine neutralizing monoclonal antibodies
Source: PLoS Pathog. 2023 Nov 20;19(11):e1011811. doi: 10.1371/journal.ppat.1011811 (PMC10695380; doi:10.1371/journal.ppat.1011811)

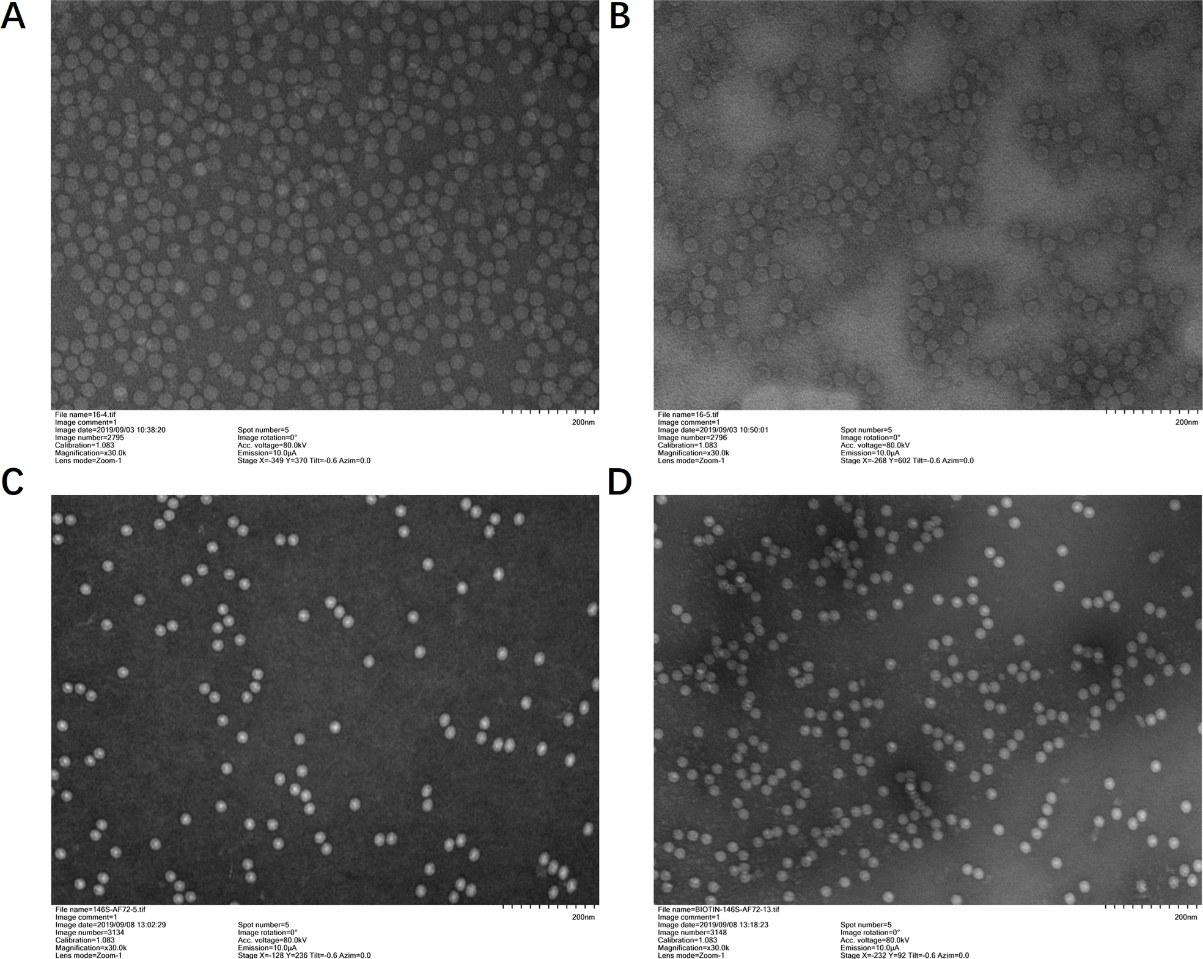

Supplement: S1 Fig — Negative strain EM analysis of purified A/GDMM/2013 146S (A) and the resulting biotinylated A/GDMM/2013 (B). Negative strain EM analysis of purified A/AF72 146S (C) and the resulting biotinylated A/AF72 (D). (TIF) [file ppat.1011811.s001.tif]

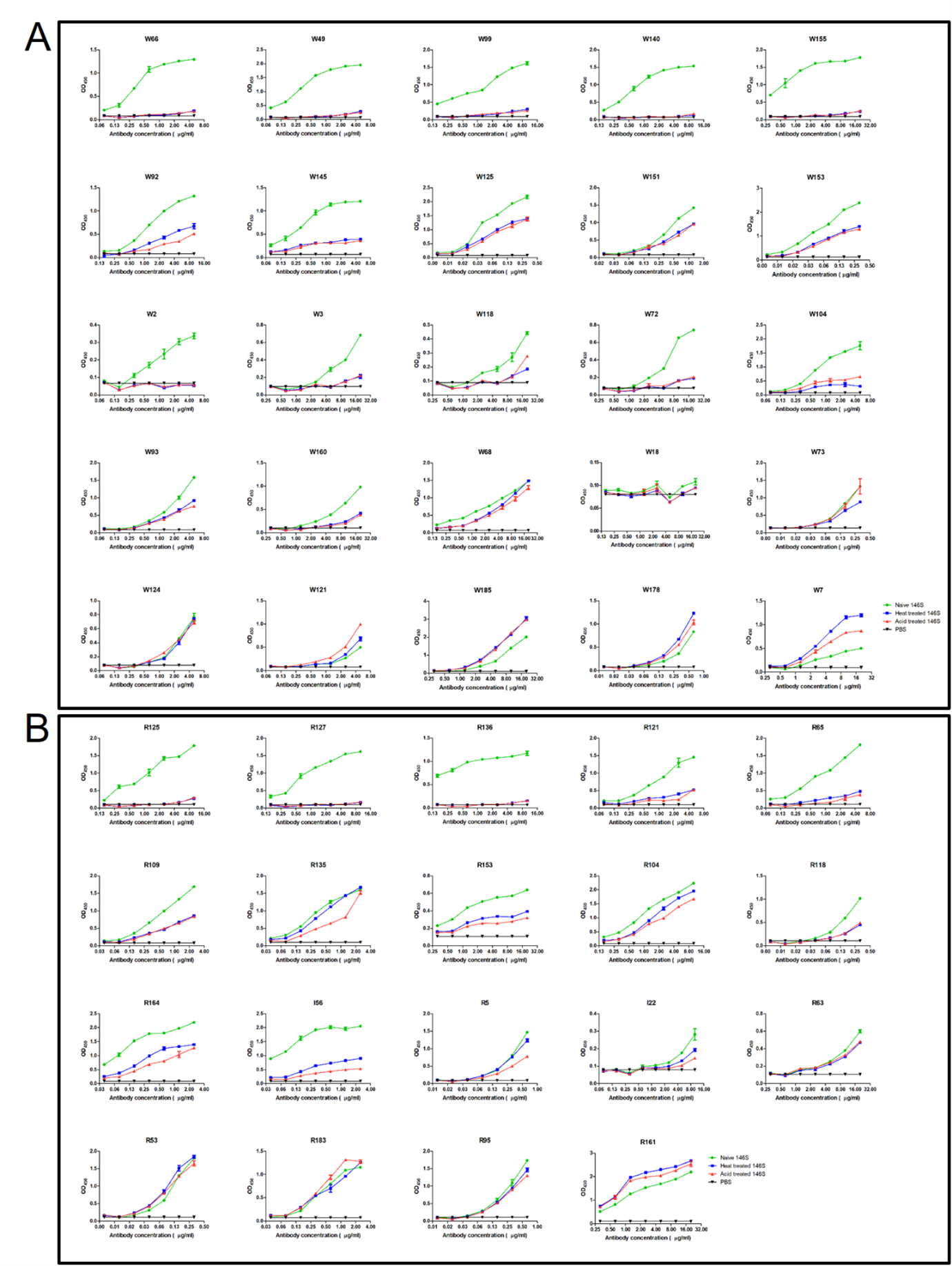

Supplement: S2 Fig — 12S particles were prepared from naïve 146S particles by acidification (incubation with NaH2PO4 (pH = 5.5) for 10 mins) or heat treatment for 1 h at 56°C. In indirect ELISA experiments, 100 ng/well of naïve 146S / acid treated 146S / heat treated 146S was respectively coated in 96-well plates overnight at room temperature. The plates were then washed three times with PBST (PBS buffer plus 0.05% Tween) and blocked with 1% gelatin in PBS at 37°C for 2 h. After three washes, the bovine mAbs at different concentrations were added and incubated at 37°C for 1 h. The plates were washed three times with PBST, and then the HRP-conjugated anti-His tag antibody (Genscript, China) at a dilution of 1:5,000 was added to the wells. The plates were then incubated at 37°C for 30 min and washed three times with PBST. Color was developed by adding 50 μl of TMB substrate (Pierce, Life Technology) for 10 min at room temperature. The process was stopped by adding equal volumes of 1 M H2SO4. Optical density at 450 nm (OD450) was measured on a microplate reader (BioRad). The results represent one of three independent assays with duplication. (TIF) [file ppat.1011811.s002.tif]

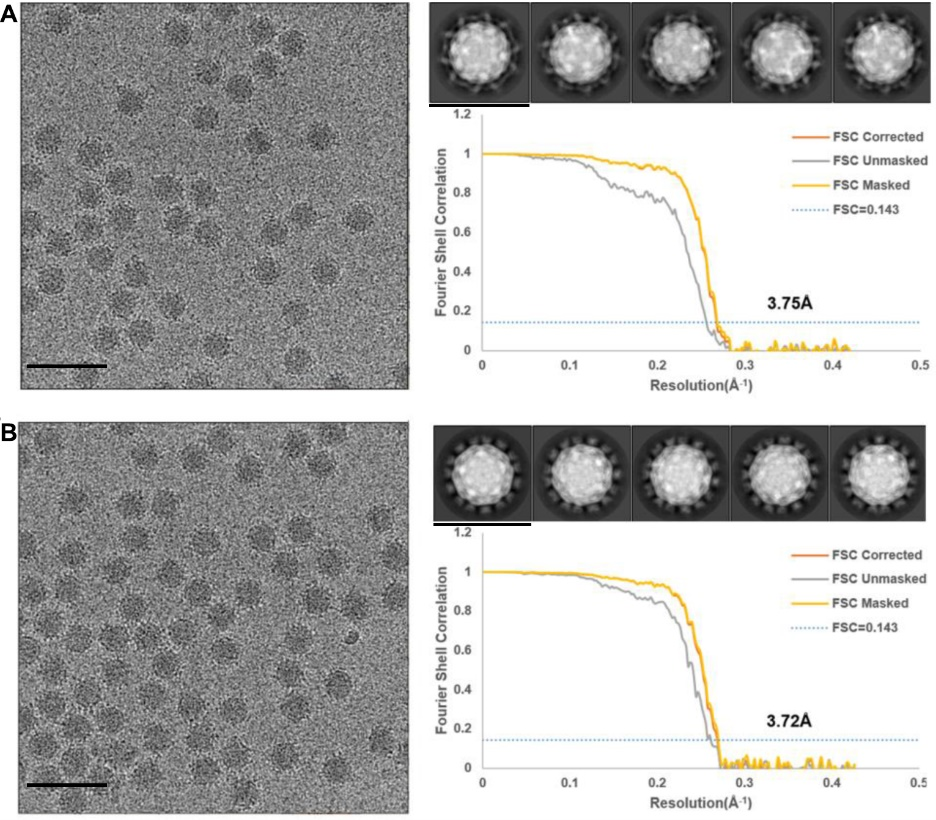

Supplement: S3 Fig — Typical electron micrographs were collected with a defocus of 1.9 μm (FMDV-AWH-W2), 1.7 μm (FMDV-AWH-W125) (Scale bar, 1000 Å). Selected 2D class averages both show prominent spikes on the outer surface of viral particles (Scale bar, 480 Å). Fourier shell correlation (FSC) of the final 3D reconstruction after gold-standard refinement using RELION and THUNDER. The resolution corresponding to an FSC of 0.143 is shown for these virus-antibody complexes. FSC curves are plotted before (gray) and after (yellow) masking in addition to post-correction (orange), accounting for the effect of the mask using phase randomization. (TIF) [file ppat.1011811.s003.tif]

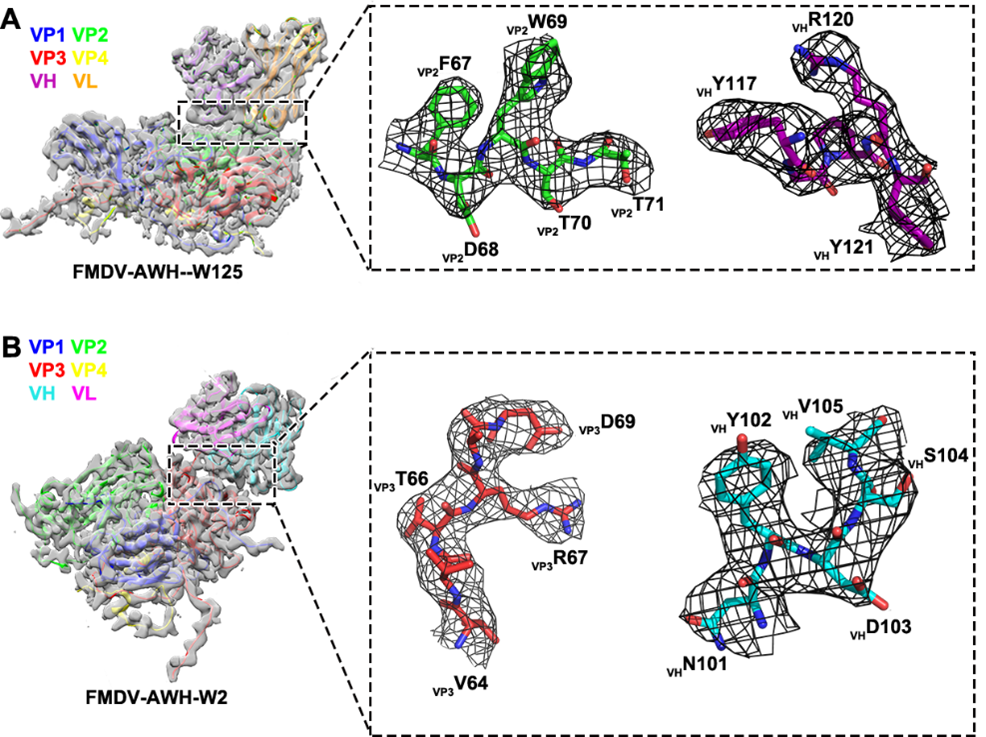

Supplement: S4 Fig — Surface representation of the density maps for a protomer of FMDV-AWH-W125 complex (A) and FMDV-AWH-W2 complex (B). VP1, VP2, VP3 and VP4 of the protomer are blue, green, red and yellow; VH and VL of W125 are purple and orange, respectively; VH and VL of W2 are cyan and magenta, respectively. In the right panel, atomic models shown as sticks are superimposed to indicate the representative regions in wire frames. In the stick models, the residue numbers are indicated. The VP2, VP3, VH and VL residues are labeled with a subscript. (TIF) [file ppat.1011811.s004.tif]

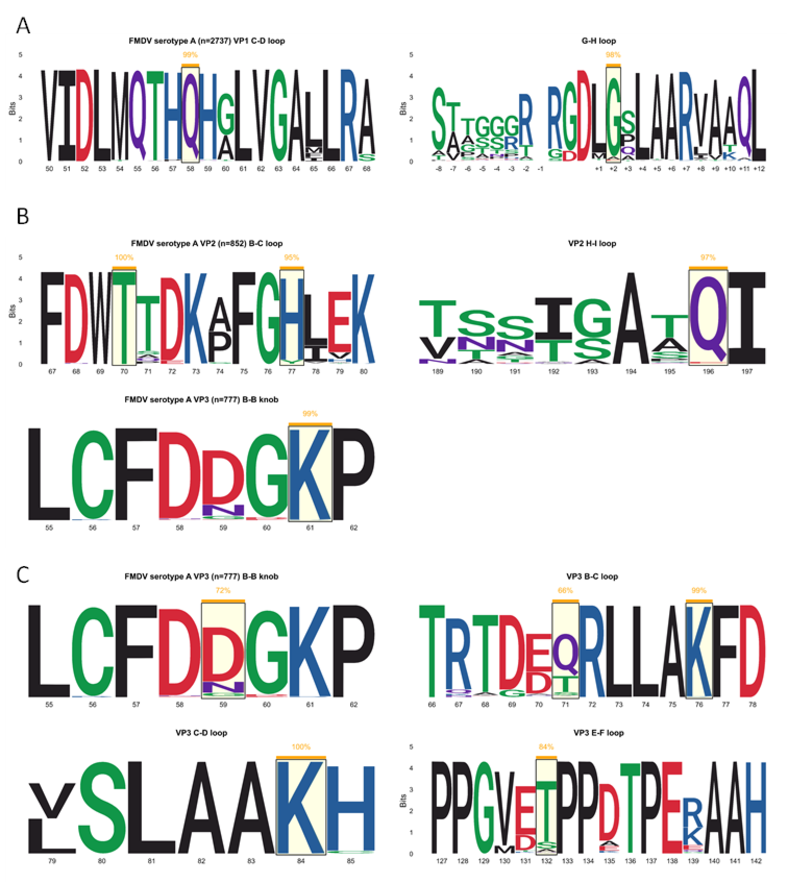

Supplement: S5 Fig — The full amino acids sequences of VP1, VP2 and VP3 of FMDV serotype A were downloaded from national center for biotechnology information (NCBI) as of June 30, 2023. The key antigenic determinants involved in common residues of A/WH/CHA/09, A/GDMM/2013 and A/AF72 were framed with rectangles and the conservation of corresponding residue was marked with orange. The key residues 58 (Q) and 147 (G, corresponding to RGD+2 position) on VP1 were determined by bnAbs W151, W153 and W145 (A). The key residues VP2 70 (T), 77 (H), 196 (Q) and VP3 61 (K) were targeted by bnAb W125 (B). The key residues 59 (D), 71 (Q), 76 (K), 84 (K) and 132 (T) on VP3 were targeted by bnAb W2 (C). (TIF) [file ppat.1011811.s005.tif]

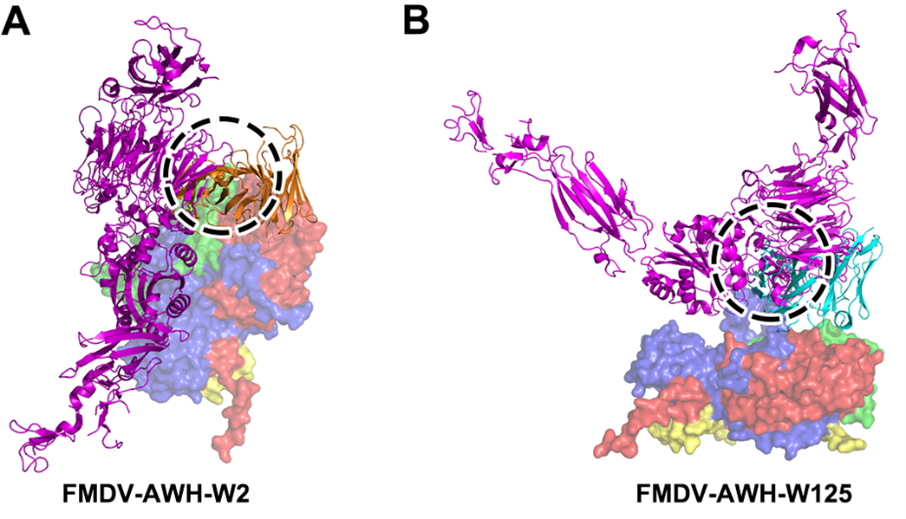

Supplement: S6 Fig — Binding modes of integrin (avβ6) receptor with scFv antibody W2 (A) and W125 (B). The panel shows a view down onto the capsid surface. VP1, VP2, VP3 and VP4 of the protomer are blue, green, red and yellow, respectively. The integrin and antibodies (W2 and W125) are drawn in cartoon representation; integrin is purple; W2 and W125 are respectively colored with orange and cyan. Black dashed circles show significant clashes between antibody (W2 and W125) and integrin receptor. (TIF) [file ppat.1011811.s006.tif]
