# Supplementary material for: Conserved antigen structures and antibody-driven variations on foot-and-mouth disease virus serotype A revealed by bovine neutralizing monoclonal antibodies
Source: PLoS Pathog. 2023 Nov 20;19(11):e1011811. doi: 10.1371/journal.ppat.1011811 (PMC10695380; doi:10.1371/journal.ppat.1011811)
Supplement: S1 Table — (DOCX) [file ppat.1011811.s007.docx]

**S1 Table. FMDV-AWH-W125 interaction residues.**

| Domain | Residue | Distance (Å) | W125 | CDR |
| --- | --- | --- | --- | --- |
| VP2 βB | D68(OD2) | 2.73 | Y117(OH) | HCDR3 |
| VP2 BC-Loop | T70(OG1) | 2.16 | Y36(OH) | LCDR1 |
|  | T71(OG1) | 3.14 | Y36(OH) | LCDR1 |
|  | K73(NZ) | 2.90 | R120(NH2) | HCDR3 |
|  | H77(NE2) | 3.28 | Y112(OH) | HCDR3 |
| VP2 EF-Loop | E131(OE1)  K137(NZ) | 3.29  3.46 | Y56(OH)  Y103(OH) | HCDR1  HCDR3 |
| VP2 HI-Loop | Q196(OE1) | 2.54 | Y117(OH) | HCDR3 |
| VP3 B-B knob | K61(NZ)  Y63(OH) | 3.63  2.72 | S97(OG)  S97(OG) | LCDR3  LCDR3 |
| VP3 HI-Loop | Q197(OE1) | 2.95 | S28(OG) | LCDR1 |

The interaction residues were computed using the CCP4 hydrogen bond distance cutoff of 4.0 Å and the salt-bridge distance cutoff of 4.0 Å.
