# Supplementary material for: Conserved antigen structures and antibody-driven variations on foot-and-mouth disease virus serotype A revealed by bovine neutralizing monoclonal antibodies
Source: PLoS Pathog. 2023 Nov 20;19(11):e1011811. doi: 10.1371/journal.ppat.1011811 (PMC10695380; doi:10.1371/journal.ppat.1011811)
Supplement: S2 Table — (DOCX) [file ppat.1011811.s008.docx]

**S2 Table. Tissue culture infective dose 50% (TCID_50_) of the rescued mutant viruses.**

| **No** | **Mutant** | **TCID_50_/ml** |
| --- | --- | --- |
| 1 | WT (A/WH/CHA/09) | 10^6.15^ |
| 2 | VP3 D59A | 10^6.0^ |
| 3 | VP3 Q71A | 10^5.0^ |
| 4 | VP3 K76A | 10^6.50^ |
| 5 | VP3 K84A | 10^6.0^ |
| 6 | VP3 T131A | 10^5.80^ |
| 7 | VP2 T132A | 10^6.10^ |
| 8 | VP3 T178A | 10^6.40^ |
| 9 | VP2 D68A | 10^5.80^ |
| 10 | VP2 T70A | 10^5.90^ |
| 11 | VP2 T71A | 10^6.90^ |
| 12 | VP2 H77A | 10^6.05^ |
| 13 | VP2 E131A | 10^7.0^ |
| 14 | VP2 Q196A | 10^6.70^ |
| 15 | VP3 K61A | 10^6.90^ |
| 16 | VP3 Q197A | 10^7.0^ |

**The TCID_50_ of rescued mutant viruses was performed on BHK-21 cells.**
