# Supplementary material for: Conserved antigen structures and antibody-driven variations on foot-and-mouth disease virus serotype A revealed by bovine neutralizing monoclonal antibodies
Source: PLoS Pathog. 2023 Nov 20;19(11):e1011811. doi: 10.1371/journal.ppat.1011811 (PMC10695380; doi:10.1371/journal.ppat.1011811)
Supplement: S3 Table — (DOCX) [file ppat.1011811.s009.docx]

**S3 Table. FMDV-AWH-W2 interaction residues.**

| Domain | Residue | Distance (Å) | W2 | CDR |
| --- | --- | --- | --- | --- |
| VP3 B-B knob | D59(OD1) | 2.88 | Y36(OH) | LCDR1 |
|  | D59(OD2) | 3.00 | Y36(OH) | LCDR1 |
| VP3 BC-Loop | R67(NE) | 3.88 | Y111(OH) | HCDR3 |
|  | R67(NH2) | 3.52 | S112(OG) | HCDR3 |
|  | R67(NH2) | 3.58 | D103(OD1) | HCDR3 |
|  | D69(OD2) | 3.00 | S104(OG) | HCDR3 |
|  | Q71(NE2) | 2.64 | Y102(OH) | HCDR3 |
| VP3 βC | K76(NZ) | 3.34 | N101(OD1) | HCDR3 |
| VP3 CD-Loop | K84(NZ) | 2.57 | N114(ND2) | HCDR3 |
| VP3 EF-Loop | T131(OG1)  T132(OG1) | 3.63  2.72 | S30(OD1)  N101(OD1) | HCDR1  HCDR3 |
| VP3 GH-Loop | T178(OG1)  T179(OG1) | 3.95  3.15 | R31(NH1)  R31(NH1) | HCDR1  HCDR1 |

The interaction residues were computed using the CCP4 hydrogen bond distance cutoff of 4.0 Å and the salt-bridge distance cutoff of 4.0 Å.
