# Supplementary material for: Conserved antigen structures and antibody-driven variations on foot-and-mouth disease virus serotype A revealed by bovine neutralizing monoclonal antibodies
Source: PLoS Pathog. 2023 Nov 20;19(11):e1011811. doi: 10.1371/journal.ppat.1011811 (PMC10695380; doi:10.1371/journal.ppat.1011811)
Supplement: S4 Table — (DOCX) [file ppat.1011811.s010.docx]

**S4 Table. Strain-specific bovine neutralizing mAb escape mutants.**

| MAb | Parent virus | Residue change | Frequency  of mutants^$^ | Neutralization  Concentration^#^  (µg/ml) | Antigenic site |
| --- | --- | --- | --- | --- | --- |
| W3 | A/AF72 | VP3 A68T  VP3 D59E, A68T  VP3 D59A, A68T  VP3 K61I, A68T  VP3 D59E, A68V, S205N | 3/7  1/7  1/7  1/7  1/7 | 600  600  600  600  600 | Strain-specific epitope (VP3 68) |
| W72 | A/AF72 | VP3 D59, A68T  VP3 A68T  VP3 V65E, A68V | 1/7  5/7  1/7 | 600  600  600 | Strain-specific epitope (VP3 68) |
| W118 | A/AF72 | VP3 A68T, Q71R  VP3 A68V  VP3 A68V, L73R | 2/5  2/5  1/5 | 600  600  600 | Strain-specific epitope (VP3 68) |
| W160 | A/AF72 | VP3 A68T, Q71R  VP3 Q71R  VP3 V65E, Q71R | 2/6  3/6  1/6 | 600  600  600 | Strain-specific epitope (VP3 68) |
| W66 | A/AF72 | VP3 Q71R  VP3 R67K | 6/8  2/8 | 600  600 | Strain-specific epitope (VP3 67) |
| W124 | A/AF72 | VP3 A68T  VP3 A68T, A119V  VP3 A8T | 4/6  1/6  1/6 | 600  600  600 | Strain-specific epitope (VP3 68) |
| W93 | A/AF72 | VP3 R67K  VP3 A68S, Q71R  VP3 A68T, Q71R | 3/5  1/5  1/5 | 600  600  600 | Strain-specific epitope (VP3 68) |
| W185 | A/AF72 | VP3 A68T  VP3 A68V  VP3 A68V; VP2 T56A | 2/5  2/5  1/5 | 600  600  600 | Strain-specific epitope (VP3 68) |
| W140 | A/AF72 | VP3 A68T, T136K  VP3 A68V  VP3 A68T; VP2 K198E  VP2 H56R  VP3 A68V; VP2 K198E  VP3 A66M; VP2 H65P | 2/8  2/8  1/8  1/8  1/8  1/8 | 600  600  600  600  600  600 | Strain-specific epitope (VP3 68) |
| W92 | A/AF72 | VP3 R67K  VP3 R67K; VP2 S185T  VP3 R67K; VP2 T71P  VP3 R67K, M130T, V202M | 4/8  1/8  1/8  2/8 | 580  580  580  580 | Strain-specific epitope (VP3 67) |
| W49 | A/AF72 | VP3 A68T  VP3 D59N, A68T  VP3 D59G, A68T  VP3 D59V, A68T; VP2 N190S  VP3 D59G, A68T; VP2 N190S | 2/6  1/6  1/6  1/6  1/6 | 600  600  600  600  600 | Strain-specific epitope (VP3 68) |
| W18 | A/AF72 | VP3 A68T, I187S  VP3 A68T  VP3 A68T, D197G  VP3 A68T, E196K  VP3 A68T; VP2 K88R  VP3 A68T; VP2 N190S | 1/7  1/7  2/7  1/7  1/7  1/7 | 600  600  600  600  600  600 | Strain-specific epitope (VP3 68) |
| W68 | A/AF72 | VP3 D59E, A68T  VP3 D59E, A68V  VP3 A68T  VP3 A68V  VP3 D59E, A68T, I94M  VP3 A68T; VP2 N190S | 1/6  1/6  1/6  1/6  1/6  1/6 | 600  600  600  600  600  600 | Strain-specific epitope (VP3 68) |
| W178 | A/AF72 | VP1 L147Q | 8/8 | 600 | Site 5 |
| W73 | A/AF72 | VP1 L147R  VP1 L150P  VP1 L147R; VP4 E49D  VP1 S149P, L150F | 3/8  2/8  2/8  1/8 | 600  600  600  600 | Site 5 |
| W7 | A/AF72 | VP1 R153G  VP1 A152V  VP1 G148E  VP1 A151E  VP1 G148E; VP2 G118C, Y138H | 3/8  1/8  2/8  1/8  1/8 | 600  600  600  600  600 | Site 5 |
| W104 | A/AF72 | VP1 H99R  VP1 H99R, Q198P  VP1 H99R, Q198A | 2/5  1/5  2/5 | 600  600  600 | Other |
| W155 | A/AF72 | VP1 Q58R  VP1 Q58R; VP2 R102K  VP1 Q58R, A151S; VP2 T8A, L134P | 3/5  1/5  1/5 | 600  600  600 | Site 3 |
| W99 | A/AF72 | VP1 Q198R  VP1 Q198R; VP2 Q196L | 1/5  4/5 | 600  600 | Site 1 |
| W121 | A/AF72 | VP2 D72A  VP2 D72N, Y171S  VP2 T71K  VP2 D72N | 2/5  1/5  1/5  1/5 | 600  600  600  600 | Site 2 |
| R118 | A/WH/09 | VP2 P74H  VP2 P74S, H77Y; VP3 A195V;  VP1 D99E;  VP2 P74S, H77Y; VP3 D177A, A195V  VP2 P74S; VP3 K61N, S114P, D174G | 1/4  1/4  1/4  1/4 | 600  600  600  600 | Site 2 |
| R5 | A/GDMM/2013 | VP2 A71T, E79K, E82K  VP2 A71T, E79K, E82K; VP3 C56R | 2/4  2/4 | 600  600 | Site 2 |
| R95 | A/GDMM/2013 | VP2 E131G, A71T, D72N, E82K  VP2 A71T, D72N | 2/4  2/4 | 600  600 | Site 2 |
| R153 | A/GDMM/2013 | VP3 D174G; VP1 K203I  VP3 A25V, E175K; VP1 T48K  VP3 A25V, E175K  VP3 A25V, E175K, D174G; VP1 T48K  VP3 A25V, E175K, D174G; VP1 T48A  VP3 A25V, E175K; VP1 A206T  VP3 A25V, E175K; VP1 R167W | 1/8  2/8  1/8  1/8  1/8  1/8  1/8 | 600  600  600  600  600  600  600 | Strain-specific epitope (VP3 175) |
| R161 | A/GDMM/2013 | VP2 K137R  VP1 P47H; VP2 K137R, Y171N  VP2 Y171N  VP2 K137R; Y171N  VP1 G46D | 1/7  1/7  2/7  2/7  1/7 | 640  640  640  640  640 | Other |
| R164 | A/GDMM/2013 | VP1 Q99R; VP3 E175K  VP1 Q99R; VP2 Y171N; VP3 E175K  VP1 Q99R; VP2 Y171K; VP3 E175K | 1/7  2/7  4/7 | 640  640  640 | Strain-specific epitope (VP3 175) |
| R183 | A/GDMM/2013 | VP3 A220V  VP3 A25V, E175K; VP1 Q99R  VP3 K61Q; VP1 A208V  VP3 A25V, E175K, A220V | 3/7  2/7  1/7  1/7 | 600  600  600  600 | Strain-specific epitope (VP3 175) |
| R136 | A/GDMM/2013 | VP3 T68A  VP3 A25V, T68A, E175K  VP3 A25V, T68M, E175K  VP3 T68M; VP1 V192A | 5/8  1/8  1/8  1/8 | 600  600  600  600 | Strain-specific epitope (VP3 68) |
| R135 | A/GDMM/2013 | VP1 K43R; VP1 A96E  VP1 K43E  VP1 K43E; VP2 Y171N  VP1 K43N; VP3 E175K  VP1 K43E; VP3 E175K  VP1 Q99R; VP3 E175K | 1/8  1/8  2/8  1/8  1/8  2/8 | 640  640  640  640  640  640 | Strain-specific epitope (VP3 175) |
| R109 | A/GDMM/2013 | VP1 G147E  VP1 G147S  VP1 P148S; VP2 Y171N  VP1 G147A; VP2 Y171N  VP1 G147S; VP2 Y171N  VP1 C146S; VP2 K130R  VP1 G147A; VP2 Y171N; VP3 E175K | 1/8  2/8  1/8  1/8  1/8  1/8  1/8 | 640  640  640  640  640  640  640 | Site 5 |
| R104 | A/GDMM/2013 | VP2 R102T; VP1 G46C  VP3 A25V, E175K; VP1 H49R  VP2 Q115P; VP1 G46S  VP1 H49Y  VP1 G46S | 1/6  1/6  1/6  2/6  1/6 | 600  600  600  600  600 | Site 3 |
| I56 | A/GDMM/2013 | VP1 G46S  VP3 E175Q; VP1 Q58K  VP3 E175K; VP1 Q58K, N141K  VP3 A25V, D174G, E175K; VP1 T48A | 1/6  1/6  3/6  1/6 | 600  600  600  600 | Strain-specific epitope (VP3 175) |
| R53 | A/GDMM/2013 | VP1 L153P  VP1 P148L  VP1 L149F  VP1 A150V; VP3 E175K  VP1 L149F; VP3 E175K  VP1 G147A; VP3 E175K  VP1 E175K; VP3 E175K | 1/7  1/7  1/7  1/7  1/7  1/7  1/7 | 640  640  640  640  640  640  640 | Strain-specific epitope (VP3 175) |
| R63 | A/GDMM/2013 | VP2 K137R; VP3 K139E | 1/1 | 600 | Other |
| R65 | A/GDMM/2013 | VP3 E175K  VP1 A121G, P122A; VP2 Q134L; VP3 E175K  VP2 Q134P; VP3 E175K  VP2 Q134L; VP3 E175K  VP2 Q134K,Y171N; VP3 E175K  VP2 Y171N; VP3 E175K  VP2 Y171N; VP3 E175K; VP1 G144S  VP2 Q134P, Y171N; VP3 E175K; VP1 G144D | 1/8  1/8  1/8  1/8  1/8  1/8  1/8  1/8 | 640  640  640  640  640  640  640  640 | Strain-specific epitope (VP3 175) |
| R121 | A/GDMM/2013 | VP3 K61R  VP3 K61Q  VP2 K130R; VP3 K61Q  VP3 A25V, E175K; VP1 Q99R  VP3 K61P  VP3 K61T  VP2 P74S | 1/8  2/8  1/8  1/8  1/8  1/8  1/8 | 600  600  600  600  600  600  600 | Strain-specific epitope (VP3 175) |
| I22 | A/GDMM/2013 | VP3 A195V; VP1 G84D  VP3 A195V; VP1 N81D  VP2 Y171N;  VP1 E170K  VP1 H172R  VP2 Y171S  VP1 N81D | 2/8  1/8  1/8  1/8  1/8  1/8  1/8 | 600  600  600  600  600  600  600 | Other |
| R125 | A/GDMM/2013 | VP3 A25V, E175K  VP3 A25V, E175K, D174A  VP3 A25V, E175K, D174G | 3/6  1/6  2/6 | 600  600  600 | Strain-specific epitope (VP3 175) |
| R127 | A/GDMM/2013 | VP2 D72E  VP2 D72E, K130R  VP2 P74S, Q196K  VP2 K73R; VP3 E175K | 2/5  1/5  1/5  1/5 | 640  640  640  640 | Site 2 |

^#^Neutralization concentration was determined as the lowest antibody concentration that protected cells from CPE.

^$^Frequencies of the mutants are the number of mutants with the mutation at the indicated residue/total number of mutants obtained.
