# Supplementary material for: Conserved antigen structures and antibody-driven variations on foot-and-mouth disease virus serotype A revealed by bovine neutralizing monoclonal antibodies
Source: PLoS Pathog. 2023 Nov 20;19(11):e1011811. doi: 10.1371/journal.ppat.1011811 (PMC10695380; doi:10.1371/journal.ppat.1011811)
Supplement: S5 Table — (DOCX) [file ppat.1011811.s011.docx]

**S5 Table. Cryo-EM data collection and refinement statistics.**

|  | **FMDV-AWH-W2** | **FMDV-AWH-W125** |
| --- | --- | --- |
| **Data collection and processing** |  |  |
| Magnification | 110,000 | 110,000 |
| Voltage (kV) | 200 | 200 |
| Electron exposure (e–/Å^2^) | 25 | 25 |
| Defocus range (μm) | -2.4 to -1.4 | -2.4 to -1.4 |
| Pixel size (Å) | 0.93 | 0.93 |
| Symmetry imposed | I1 | I1 |
| Initial particle images (no.) | 21999 | 12646 |
| Final particle images (no.) | 8580 | 7054 |
| Map resolution (Å)  FSC threshold | 3.75  0.143 | 3.72  0.143 |
| Map resolution range (Å) | 3.2-4.8 | 3.2-4.8 |
|  |  |  |
| **Refinement** |  |  |
| Initial model used (PDB code) | 1BBT;  6e9u | 1BBT;  6e9u |
| Model resolution (Å)  FSC threshold | 2.60;2.29  0.143 | 2.60;2.29  0.143 |
| Model resolution range (Å) | ꝏ to 2.60 | ꝏ to 2.60 |
| Map sharpening *B* factor (Å^2^) | -199 | -189 |
| Model composition  Non-hydrogen atoms  Protein residues  Ligands | 6848  898  0 | 6944  909  0 |
| *B* factors (Å^2^)  Protein  Ligand | 13.24  -- | 25.14  -- |
| R.m.s. deviations  Bond lengths (Å)  Bond angles (°) | 0.007  0.898 | 0.010  1.010 |
| Validation  MolProbity score  Clashscore  Poor rotamers (%) | 2.33  5.14  0.00 | 2.52  6.04  0.00 |
| Ramachandran plot  Favored (%)  Allowed (%)  Disallowed (%) | 92.40  7.60  0.00 | 90.37  9.63  0.00 |
