# Supplementary material for: Conserved antigen structures and antibody-driven variations on foot-and-mouth disease virus serotype A revealed by bovine neutralizing monoclonal antibodies
Source: PLoS Pathog. 2023 Nov 20;19(11):e1011811. doi: 10.1371/journal.ppat.1011811 (PMC10695380; doi:10.1371/journal.ppat.1011811)
Supplement: Attachment — Submitted filename: Responses to comments.docx [file ppat.1011811.s012.docx]

Dear editors and reviewers:

Thank you for your letter and for the reviewers’ comments concerning our manuscript entitled “Conserved antigen structures and antibody-driven variations on foot-and-mouth disease virus serotype A revealed by bovine neutralizing monoclonal antibodies”. Those comments are all valuable and very helpful for revising and improving our paper, as well as the important guiding significance to our researches. We have studied comments carefully and have made correction which we hope meet with approval. All Figures have been checked and adjusted by PACE digital diagnostic tool. Revised supplements data were marked in yellow in the document of supporting information. The main correction in the paper and the responds to editors and reviewers’ comments are as following.

Best wishes

Zengjun Lu

Response to comments

Part I – Summary

Please use this section to discuss strengths/weaknesses of study, novelty/significance, general execution and scholarship.

Reviewer #1:

This study contains a lot of well-presented data, interesting information and proof-of-principle for how antigenic information in FMDV could potentially be used to engineer antigens capable of inducing more broadly reactive responses. If anything the significance of this is underplayed. There is scope to improve presentation of some of the rationale (suggestions elsewhere in this review). Cattle were vaccinated with a trivalent O serotype vaccine and then sequentially boosted with three separate A serotype vaccines A/WH/CHA/09, A/GDMM/CHA/2013 and A/AF72. A/AF72 is a long-established vaccine strain related to A22, A/WH/CHA/09 is a vaccine strain of the Sea97 lineage and A/GDMM/CHA/2013 is a more recent strain also from the Sea97 lineage. The introduction explains these strains but nowhere in the abstract/summary or intro is it made clear that these are being used to immunize the cattle. Presumably these strains represent wide antigenic diversity within serotype A viruses and sequential immunization with these was hoped to stimulate cross reactive responses against conserved epitopes (this rationale was not immediately clear).

Then A/AF72 and A/GDMM/2013 were used as antigenically diverse serotype A antigens to select reactive mAbs from vaccinated cattle. The rationale here was clear from the start but how are these viruses antigenically diverse, is it possible to explain this somewhere? Were these two antigens used because they were the most divergent (e.g. in contrast A/WH/CHA/09 was considered to be in the middle?)?

Responses：

Thanks for your questions and advice. There are three representative strains of FMDV type A viruses emerged in different history period, A/AF72 was the oldest strain isolated from bovine in 1972, and this strain had been used to produce inactivated vaccine in China because of its highly immunogenicity. A/WH/CHA/09 and A/GDMM/2013 were the two most recent introduced FMDV type A viruses in the 21st century belongs to Southeast Asia 97 lineage (Sea97), which occurred in 2009 and 2013, respectively. All this information had been supplemented in the abstracts and introduction of paper. Sequential vaccination with different virus strains is expected to expand the cross-neutralizing antibody responses, so that we can analyze the conserved and variable antigenic sites.

Reviewer #2

In this study by Li et al, the authors aimed to understand the structures of conserved and variable antigenic sites on foot-and-mouth disease virus (FMDV) particles. The work specifically investigates serotype A FMDV, generally considered to be one of the most antigenically diverse serotypes, however, the sites/structures of all variable and conserved antigenic sites are not fully resolved. Using two serotype A strains (one historical and one current) as bait antigen the authors identify and characterise 39 strain specific and 5 broadly neutralising antibodies. Using cryo-EM two conserved antigenic sites were proposed that involve a complex of residues on viral proteins VP1, VP2 and VP33. In contrast, stain specific neutralising antibodies involved more key residues in VP3 that were more variable between strains. The authors proposes VP3 and VP1 are more flexible with VP2 being more structurally rigid, which could drive greater broadly neutralising antibody production.

Foot-and-mouth disease virus is an economically important pathogen. Vaccines are likely key for future global control of infection and therefore understanding antigenicity is a relevant area of study. The authors present a commendable amount of data that convincingly characterised specific and more broadly neutralising antigenic sites of FMDV capsids. This work therefore incrementally advances the knowledge on FMDV antigenicity. With some exceptions (as described below) the data is largely presented well and is well interpreted but it does not support the overall conclusion on viral structural flexibility and the manuscript contains multiple wording, spelling or grammatical errors. Specific points to improve the study are listed below:

Responses:

Thanks for the comments. We agree with the reviewer the viewpoints that the data presented in this paper cannot fully resolve the antigenic structure of type A virus strains involved because of the complexity of antibody responses and limited No. of B cell antibodies used. However, we think this paper accumulated important data to fully understand the antigenic structure of FMDV type A. As for structural flexibility, this statement may be not accurate so we change this description to “more variable” for VP3 and VP1 based on the observation of mutations under neutralizing antibodies.

Part II – Major Issues: Key Experiments Required for Acceptance

Reviewer #2:

Question 1.

Line 391: The authors use 146S antigen for immunisation. The production/purification of the antigen was not described. How can you ensure it is not contaminated with other FMDV particles that would be immunogenic (e.g., 45S particles).

Responses：

Thanks for your question. We supplemented contents in the material and method section. The fractions were analyzed by negative-stain electron microscopy, and the fraction containing 146S particles was transferred to a 100-kDa MWCO centrifugal filter for buffer exchange with PBS to remove the sucrose. The final obtained 146S antigen and the resulting biotinylated FMDV 146S antigen were all checked using negative strain electron microscope (EM) and found to be integrity before and after biotinylation. The negative strain EM results were shown in the supplemental Fig.S1.

Question 2.

Following on from point 1, do the bovine monoclonal antibodies identified recognise a 75S, 45S particle or 12S subunit, or do these particles generate the same antibodies. Understanding this is very important to understand if non-infectious particles/VLPs/antigens could illicit an appropriate immune response to be used as a vaccine candidate.

Responses：

Thanks for your questions. The 146S particles were used in this study, we did not distinguish 146S and 75S (VLPs) or 45S particles in this study. We are focusing on antigen structure but not on vaccine candidate. So, we use 146S particles as antigen to vaccinate animals because 146S particles contain all antigenic sites that can elicit antibody response. These bovine monoclonal antibodies all recognize the viral capsid proteins, which relies on structural integrity or not. To clarify the structural features recognized by bovine neutralizing antibodies, we first prepared 12S subunits via disassembly of integrated 146S particle with acid and heat separate treatments, then the obtained 12S subunits and naïve 146S particle were probed by these bovine monoclonal antibodies using indirect ELISA. As shown in Fig. S2, most of the bovine mAbs (34/44) are more bent to recognize the intact 146S particle than 12S subunits, and part of them (W66, W49, W99, W140, W155, R125, R127 and R136) showed the binding with FMDV antigens in a strictly 146S-dependent way. Whereas minority of the bovine antibodies (10/44) can recognize both 12S and 146S antigens in similar binding activity. Generally, the above results suggest the maintaining integration of FMDV 146S particle was critical to induce neutralizing antibody response in bovine, although the 12S subunit binding antibodies were identified. We make statement as the supplemental data in the first result section.

Question 3. Figure 3E/4E showing individual single IF images does not show recovery of mutant viruses convincingly, in particular since such a high MOI (10) was used for these experiments. A titre of the recovered mutant viruses would be more convincing and inform on fitness costs of these mutations. Understanding the fitness of these mutant viruses is essential for understanding the antibody neutralisation results.

Responses：

Yes, we determined the tissue culture infective dose 50% (TCID50) of the rescued mutant viruses and the results were listed in the supplemental Table 2. The TCID_50_ of the recovered mutant viruses was similar to that of the corresponding wild strain (A/WH/CHA/09), suggesting the successful recovery of mutant viruses.

Question 4. Lines 271-274: I agree the author’s data suggests that VP3 is more readily able to change sequence under immune pressure, however the data presented does not investigate structural flexibility. The authors study does not look at structural dynamics/movements of VP2 or VP3/1 but sequence tolerance/variability and ability of these proteins to sample sequence space under immune pressure. The authors should modify the wording of their conclusions here and elsewhere in the discussion.

Responses：

Thanks for your suggestion. We agree to the comments. we have modified the wording of description and please see them in the main text.

Part III – Minor Issues: Editorial and Data Presentation Modifications
Please use this section for editorial suggestions as well as relatively minor modifications of existing data that would enhance clarity.

Reviewer #1:

Question 1:

Abstract - Why not include mention of A22 and SEA97 lineages in the abstract? Why not make clear how the animals have been immunized?

Responses：

Yes, thanks for questions. As per your suggestion, we have added the description in the abstract section “Here, a historical A/AF72 strain from A22 lineage and a latest A/GDMM/2013 strain from Sea97 lineage were respectively used as bait antigen to screen single B cell antibodies from bovine sequentially vaccinated with A/WH/CHA/09, A/GDMM/2013 and A/AF72 (A22 lineage) antigens”.

Question 2

The introduction is pretty short.

After reading abstract, author summary (and introduction) I still did not understand what the cattle were immunised with. This information only seems to be provided in the methods. Give that so much emphasis is placed on describing the diverse bait antigens it feels strange that no rationale is given for the immunisation strategy. If the objective of the study was to isolate mAbs which were broadly cross reactive within the A serotype, why were the cattle first immunised with O serotype vaccines? Was this a single trivalent O vaccine? Then three A serotype vaccines are given as separate sequential boosters. Please explain the rationale for the immunisation in the results and at least mention something in the abstract. Could the initial immunisation with O serotype vaccines also influence the response to the later A vaccines due to ‘original antigenic sin’ which may constrain the direction of responses? Perhaps worth a mention in the discussion?

Responses：

Thanks for this question. In this study, the bovine used for isolation of serotype A-specific B cells was the continuation of our previous project where the bovine was sequentially immunized with three strains in FMDV serotype O (inactivated O/Mya/98, O/HN/CHA/93, and O/Tibet/99) to induce broadly neutralizing antibodies against FMDV serotype O strains (Frontiers in immunology, Kun et al, 2021). Under this backgrounds, the bovine was further sequentially immunized with inactivated FMDV A/WH/CHA/09, A/GDMM/CHA/2013 and A/AF72 at 28-day intervals. Given to this, we are not sure whether the “original antigenic sin” may disturb the direction of response to serotype A because of initial immunization with serotype O antigens, which may be a factor of uncertainty in the interpretation of the data. However, according to our unpublished data of porcine-derived monoclonal antibodies, sequential immunization benefits to obtain broadly neutralizing antibodies against FMDV, and sequential vaccination with serotype O and A can induce cross-serotype neutralizing antibodies against Asia 1 strain.

Question 3

Was any other data generated to confirm that the immunisations had worked as expected? ELISA, VNT on resulting sera?

Responses：

Dear Reviewer, to check whether immunizations had worked as expected, we performed the across virus neutralization test (VNT) of resulting sera with A/WH/CHA/09, A/GDMM/CHA/2013 and A/AF72 strains respectively. We found the bovine sera after final immunization can neutralize all three strains of serotype A with neutralizing antibody titer of >1:1024.

Question 4

For the selection of mAbs recognising conserved epitopes would it have been useful to attempt to find B cells sorted by labelling with both A/AF72 and A/GDMM/2013 antigens?

Responses：

Dear Reviewer, yes, it’s an elegant strategy to isolate cross-reacted antibody by introduction of two different bait antigens together. When labeling antigen, small molecule suitable fluorescein with a small molecule weight (such as biotin, pacific blue etc) and maintain the integrate of 146S are two essentials for obtaining expected results.

Question 5

The Fab-virus structures are generated with A/WH/CHA/09, it would be useful to explain why this was done when none of the B cells were selected against this antigen.

Responses：

Dear Reviewer, two mAbs selected to resolve the structure are wide neutralizing antibodies against three type A virus strains, so any one of which can be used to resolve the complex structure. Contrastively, A/WH/CHA/09 strain had been evaluated to be good immunogenicity and particle stability, and currently used as a vaccine strain to prevention serotype A viruses. So, A/WH/CHA/09 was selected for resolution of antibody-virus complex structure, hoping to shed light on vaccine molecular design.

Question 6

Specific comments on language use.

2 “Ancient”: I’m not sure what this is supposed to mean but I don’t think a virus strain from 1972 qualifies as ancient. Is this the best phrase to use?

Responses:

Thanks for your suggestions. We use “historical” instead of “ancient”.

Question 7

28 “sole VH of W2”: what does this mean? There was no mention of this detail for W125 (line 26) so is the situation with W2 something special or unusual?

Responses:

We mean it is only the VH, not VL of W2 that contacts with the viral antigen. Contrastingly, both VH and VL of W125 that were involved in the recognition of viral antigen. We had modified the content.

Question 7

0-32 Not sure if I understand this section but I think “The highly conserved epitopes…” would make more sense if it read “Additional highly conserved epitopes…”

Response:

Thanks for the good advice. As per your suggestion, we modified the word.

Question 8

4 Statement “In China, there are three representative strains of FMDV serotype A…”. Please clarify what this means. The sentence at the end of the paragraph suggests these are intended to represent antigenic diversity or antigenic evolutionary history of A serotype viruses in China… it would be useful to indicate this at the start of the paragraph.

Responses:

Yes, we mean that there are three different time representative strains of FMDV serotype A documented in China, which are good sources to reveal antigenic diversity or antigenic evolutionary history of A serotype viruses. For clarity, we indicate this at the start of the paragraph.

Question 9

37 I don’t think W125 and W2 have been introduced in the main text yet.

Response:

We added the background of bnAbs W2 and W125 at the start of the paragraph in main text. Please see it as follows.

“Less information about conservative antigen structure on the VP2 and VP3 of FMDV serotype A was available. Neutralization escape mutants suggested the recognition of bnAbs W125 and W2 was involved in VP2 and VP3.”

Question 10

152/153 what do “CCP4” and “RIVEM” stand for? LCDR?

Responses:

CCP4 is a software suite, which can be used to analyze the protein-protein (viral capsid-antibody) interactions. RIVEM is a computer program, which can be used to generate a spherical roadmap of FMDV surface residues. LCDR indicates the complementary determining region of light chain for an antibody molecule.

Question 11

387 “…basic immunization with the three topotypes of FMDV serotype O…” what does basic mean? Was this a single trivalent vaccine or separate sequential vaccines?

Responses:

It is not a single trivalent vaccine, and we carried out the separate sequential vaccines. For clarity, we made the modification for this paragraph. See it as follows.

“Briefly, bovine have a basic immunization with the separate three topotypes of FMDV serotype O (inactivated O/FMDV/Mya/98, O/HN/CHA/93, and FMDV O/Tibet/99), as described in our previous report. Then the bovine, designed as #1217, was further sequentially immunized with the inactivated vaccines of FMDV A/WH/CHA/09, A/GDMM/CHA/2013 and A/AF72 at 28-day intervals.”

Question 12

Various other examples where wording or language could be improved. Please check.

Response：

Yes, we had carefully read and improved the language and wording.

Question 13

Reviewer #2:

1. In several places (e.g., figures 3G, 4G and lines 173-174) the authors state amino acids are “highly conserved” but only align the three sequences used in this study. Making reference to figure 5 where a larger alignment is conducted would offer more insight on the conservation of the residues investigated.

Responses：

To check the conservation of the residues investigated, we surveyed all the FMDV sequences (n=2373) of serotype A available from NCBI until June 30, 2023. Alignment of amino acids of VP1 (n=2373) revealed the VP1 58 (Q) and 147 (G; corresponding to the RGD +2 position) residues were highly conserved in isolates of FMDV serotype A, showing conservation of 99% and 98% separately. For the key antigenic determinants targeted by W125, alignment of amino acids of VP2 (n=852) as well as that of VP3 (n=777) revealed the involved common residues of A/WH/ CHA/09, A/GDMM/CHA/2013 and A/AF72 were highly conservative, showing conservation of 100%, 95%, 97%, and 99% for VP2 70 (T), 77 (H), 196 (Q) and VP3 61 (K), respectively. For the key antigenic determinants targeted by W2, alignment of amino acids of VP3 (n=777) revealed the involved two common residues (VP3 76 (K), 84 (K)) were extremely conservative, showing conservation of 99% and 100%, respectively. The remaining three residues (VP3 59 (D), 71 (Q) and 132 (T)) showed a slightly low conservation with 72%, 66% and 84%, respectively. These data were presented in supplements of FIG.S5.

Question 14

2. I feel the abstract contains too much detail/assumed information (e.g., long list of amino acid interactions and names of viral proteins). To make it accessible to the broader readership I would consider changing the abstract to make it more accessible to non-FMDV experts.

Responses：

Dear Edit, thanks for your suggestion. We delete the detail interactive residues and try to change the abstract for accessible to non-FMDV experts. Please see it in the main text.

Question 15

3. Lines 142-143: The authors conclude a total of 60 scFv were bound to each virion. Using icosahedral averaging for the cryo-EM reconstructions can the authors be sure of this occupancy?

Responses：

During Cryo-EM sample preparation and data collection, the FMDV 146S and scFv were incubated at a molar ratio of 1:240 in a volume of 50 µl (see material and methods section), and this will make sure that the epitopes in virus capsid were fully covered by scFv.

Question 16

4. Lines 20-21: please include additional references to support the antigenic diversity of serotype A FMDV and previous work on FMDV serotype A antigenicity such as PMID: 36699337.

Responses：

It is generally acknowledged that serotype A was the most antigenic diversity. To further confirm the opinion, we downloaded all the available FMDV serotype O, A and Asia 1 sequences from NCBI. Using MEGA software, we computed the overall mean distance of VP1 amino acids for the serotype A (n=2737), O (n=6265) and Asia1 (n=824), respectively. As reveled in the results, the overall mean distance of serotype A is 12.8%, obvious higher than serotype O (8.4%) and Asia1(7.8%). We checked the work (PMID: 36699337) and found it is more associated with the 146S specific epitope. So, we added this reference in the first part of result section. Please see it in the main text.

Question 17

5. Lines 54-55: there have been no reports of serotype C FMDV since 2004 and it is now considered to be extinct. Please modifying the wording.

Responses：

Yes, we correct the description in the introduction .

Question 18

6. Lines 71-72: please include references for the identification of antigenic sites based on murine antibodies.

Responses：

Yes, we added the references on murine antibodies.

Question 19

7. Lines 111-112: “the proportions of A/AF72-binding B cells was 0.06%”. I cannot see how this number is calculated from tables 1F or 1G.

Responses：

The proportion of antigen-binding class-switched B cells is the cell counts of gate P4 divided by cell counts of gate P1. The proportion of A/GDMM/2013-binding B cells (gated P4) is 0.06% (606/1097942) of viable PBMCs (gated P1) and the proportion of A/AF72-binding B cells (gated P4) is 0.05% (=663/1376856) of viable PBMCs (gated P4). So, we corrected the value.

Question 20

8. Lines 120: define VNT

Responses：

Yes, we defined the VNT in the content.

Question 21

9. Figure 1: some panels of data could be relocated to supplementary to streamline the figure, such as panels A-C.

Responses：

The main aim of displaying panels A-C was to show the importance of this gating strategy for successful obtainment of specific B cell derived antibody. Due to the non-specific binding characteristic (or lower affinity) of IgM^+^ B cells, we should exclude this population and this point is very important.

Question 22

10. Figure 3A has become distorted (i.e., out of aspect ratio) and is therefore hard to interpret.

Response：

Yes, we corrected panel A in Figure 3.

Question 23

11. Line 158: should refer to figure 3D not 2D.

Response：

Yes, we corrected this.

Question 24

12. Lines 169-204 in references to figures 3/4 and lines 261-296 in reference to figure 7: it is confusing to refer to the panels of data out of order. i.e., the text refers to figure 7E before 7A. It would be easier to follow if the panels of data are in the same order as they are referred to in the text.

Response：

Yes, we adjusted the order of panels in Figure 3, 4 and 7, and their legends.

Question 25

13. Lines 449-456: how many particles were collected in the collection and used for the cryo-EM reconstructions.

Responses：

For FMDV-AWH-W2 complex, a total of 21999 particles were collected; 9406 particles were used for 3D reconstruction. For FMDV-AWH-W125 complex, a total of 12646 particles were collected; 7054 particles were used for 3D reconstruction. The data were listed in Supplemental Table 5.
