# Supplementary material for: Conserved antigen structures and antibody-driven variations on foot-and-mouth disease virus serotype A revealed by bovine neutralizing monoclonal antibodies
Source: PLoS Pathog. 2023 Nov 20;19(11):e1011811. doi: 10.1371/journal.ppat.1011811 (PMC10695380; doi:10.1371/journal.ppat.1011811)
Supplement: Attachment — Submitted filename: Letter to editor.docx [file ppat.1011811.s013.docx]

Dear editors,

Thanks very much for checking the revision and advices for improvement manuscripts. As per suggestions of reviewer 1, for the responses Q2 and Q3, we acknowledge the limitations of immunization procedure in this work, so we add the statements in the second paragraph of the discussion. “In this work, there are two limitations to the immunization schedule for accurately evaluating the antibody response to FMDV serotype A in bovine. First, regarding the potential for “original antigenic sin”, previous sequential immunizations with O serotype antigens in bovine may interfere with the quality and magnitude of neutralizing antibodies responses against serotype A. Second, this study lacks the sera data collected from different times after sequential immunizations with A antigens, so we cannot rule out that only one of the three immunizations were effective and that the sera simply cross-reacted to neutralize all three serotype A strains.”

For response to Q5, we added the explained information as follows. “The two bnAbs can effectively neutralize all the three serotype A strains. Contrastively, A/WH/CHA/09 strain had been evaluated to be good immunogenicity and particle stability, and currently used as a vaccine strain to prevention serotype A viruses. Thus,…”.

For response to Q11, we modified the text as per suggestion.

Additionally, all the figures had been checked by PACE and the references were also checked. The manuscript was meticulously revised and polished by the authors to enhance its clarity, coherence, and readability, thereby hoping to meet the stringent standards of your professional and academic publication. Thanks again!

Best wishes,

Zengjun Lu
